# A 3D-Printed Piezoelectric Microdevice for Human Energy Harvesting for Wearable Biosensors

**DOI:** 10.3390/mi15010118

**Published:** 2024-01-10

**Authors:** Ihor Sobianin, Sotiria D. Psoma, Antonios Tourlidakis

**Affiliations:** 1 School of Engineering & Innovation, The Open University, Walton Hall, Milton Keynes MK7 6AA, UK; ihor.sobianin@open.ac.uk (I.S.); sotiria.psoma@open.ac.uk (S.D.P.); 2 Department of Mechanical Engineering, University of Western Macedonia, 50100 Kozani, Greece

**Keywords:** wearable biosensors, human energy harvesting, piezoelectric nanogenerator, computational fluid dynamics (CFD), piezoelectricity, arterial pressure, 3D printing

## Abstract

The human body is a source of multiple types of energy, such as mechanical, thermal and biochemical, which can be scavenged through appropriate technological means. Mechanical vibrations originating from contraction and expansion of the radial artery represent a reliable source of displacement to be picked up and exploited by a harvester. The continuous monitoring of physiological biomarkers is an essential part of the timely and accurate diagnosis of a disease with subsequent medical treatment, and wearable biosensors are increasingly utilized for biomedical data acquisition of important biomarkers. However, they rely on batteries and their replacement introduces a discontinuity in measured signals, which could be critical for the patients and also causes discomfort. In the present work, the research into a novel 3D-printed wearable energy harvesting platform for scavenging energy from arterial pulsations via a piezoelectric material is described. An elastic thermoplastic polyurethane (TPU) film, which forms an air chamber between the skin and the piezoelectric disc electrode, was introduced to provide better adsorption to the skin, prevent damage to the piezoelectric disc and electrically isolate components in the platform from the human body. Computational fluid dynamics in the framework of COMSOL Multiphysics 6.1 software was employed to perform a series of coupled time-varying simulations of the interaction among a number of associated physical phenomena. The mathematical model of the harvester was investigated computationally, and quantification of the output energy and power parameters was used for comparisons. A prototype wearable platform enclosure was designed and manufactured using fused filament fabrication (FFF). The influence of the piezoelectric disc material and its diameter on the electrical output were studied and various geometrical parameters of the enclosure and the TPU film were optimized based on theoretical and empirical data. Physiological data, such as interdependency between the harvester skin fit and voltage output, were obtained.

## 1. Introduction

It is important to timely detect any anomalous deviations of the human body physiological biomarkers to prevent further deterioration of health. To achieve this, various biosensors are employed to continuously monitor the health condition of patients [1,2]. However, this functionality is usually impaired by the use of batteries [3]. When a battery depletes, the procedure of its replacement implies that a discontinuity in measurements occurs. Moreover, glucose sensors in particular have to penetrate the skin with a needle and this process repeats itself any time the sensor needs to be replaced, which brings pain and discomfort to its user. One way of addressing these challenges is energy harvesting from the human body [4,5].

The human body is a source of multiple types of energy. Namely, biochemical, thermal and biomechanical energies can all be scavenged with energy harvesters [6,7,8]. Biomechanical energy is the largest group, which is represented by piezoelectric, triboelectric, electrostatic and electromagnetic nanogenerators [9,10]. A very reliable source of mechanical displacement is the human heart. The radial artery quite frequently is exploited for energy harvesting purposes since the collected energy can be used straight away for powering smartwatches, fitness trackers, heart rate monitors, etc. [11,12,13,14]. With the recent advances in the use of neural networks for signal processing [15], the added cost of power required for computations may be negated by extracting energy from the target sensing source, which also can allow for computations to happen with the sensor on-board electronics.

Piezoelectricity is widely used to scavenge energy from the human body due to the straightforward nature of energy generation, the low cost of material fabrication methods and the availability of a wide variety off-the-shelf components. Materials that are used for such piezoelectric nanogenerators (PENGs) are diverse and can include polymers (PVDF, PVDF-TrFE) [16], ceramics (PZT) and materials of biological origin [17,18]. These materials can also be found in a hydrogel form to enhance flexibility and stretchability and to add properties to the material such as self-healing [19]. The energy can be harvested from constant sources such as the cardiovascular and respiratory systems [20,21], but it can also originate from movement, which comes in a form of motion of joints [22] and jogging [23]. These energy harvesters can, respectively, be integrated into masks, wrist-mounted biosensors, watches, footwear, backpacks, etc. The efficiency of energy harvesting applications can increase via hybridization of PENGs and other generators. This design approach is represented by integrating the PENG with triboelectric [24], thermal [25] and electrostatic nanogenerators [26].

In the present work, an investigation of energy harvesting from the radial artery pulsations via a novel piezoelectric platform is carried out. In the proposed concept, it was found that it is critical to introduce an air chamber in between an elastic film and a piezoelectric nanogenerator (PENG) to electrically isolate the energy harvester from the body and protect the PENG from body fluids damaging it. During the systole, the artery expands and compresses the chamber, which in turn actuates the PENG. A computational model of the harvester was designed and analyzed in the COMSOL Multiphysics 6.1 software. The validity of the proposed model was verified through the comparison of the computational data with the experimental results that were obtained. An enclosure and elastic film for the PENG were manufactured with an Ultimaker S5 3D printer with filament fabrication method (FFM), thus demonstrating the simplicity and low cost of the required process. Various 3D printer settings were considered and compared in order to optimize the print quality and reduce the film thickness. Finally, an experiment involving acquisition of physiological data from a 3D-printed enclosure was carried out and assessed.

## 2. Simulations

### Working Principle of the Harvester

The cardiac cycle is characterized by diastolic and systolic phases. Diastole is a process of ventricular relaxation and filling while systole is a process of ventricular contraction which forces blood into arteries [27]. When blood is ejected from the left ventricle, it travels through the aorta down to brachial and then radial arteries. Systole and diastole are both phases that cause arteries to expand and contract, respectively, and this displacement can be scavenged for energy harvesting purposes.

**Figure 1:**
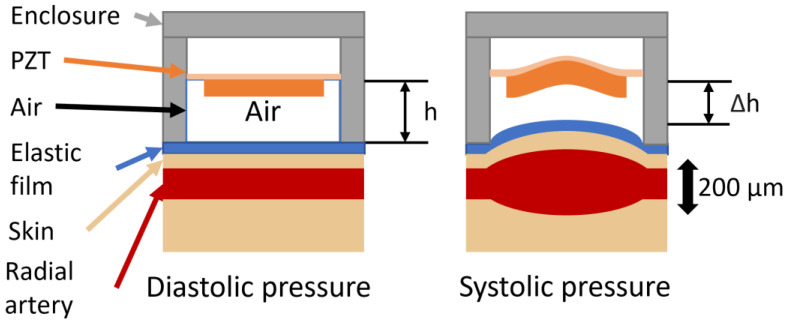
A piezoelectric energy harvesting platform operation concept.

The working principle of the developed energy harvesting platform is based on air compression inside a chamber and the subsequent excitation of a PENG, as illustrated in Figure 1. The central part of the platform is a piezoelectric disc, which is harnessed by an enclosure. In order to electrically isolate the PENG from the body and to prevent body fluids getting into the enclosure [28], it was found that an elastic film should be introduced. The resultant chamber is filled with air which is being compressed when the radial artery expands. The compression raises pressure inside the chamber and it is transferred to the PENG surface, where it can be harvested. Furthermore, even if the harvester is positioned with an offset from the radial artery, due to the relatively high coverage area, pulsations will still actuate the PENG. While some harvesters and sensors use arrays of pressure-sensitive materials [15,29], a one-piece PENG can be used with the proposed energy harvesting method.

The physical model of the energy harvesting platform is described by Newton’s Second Law:

(1) $$F=(p_{ch}-p_{0})A$$

(2) $$m\frac{d^{2}x}{{dt}^{2}}=(p_{ch}-p_{0})A$$

where *F* is the force that is applied to the film (N), $p_{ch}$ is the pressure inside the chamber (Pa), $p_{0}$ is the ambient pressure (Pa), *A* is the cross-sectional area of the film that is being displaced (m^2^), m is the mass that imposes the load (kg), *x* is the film displacement (m) and *t* is the time (s).

**Figure 2:**
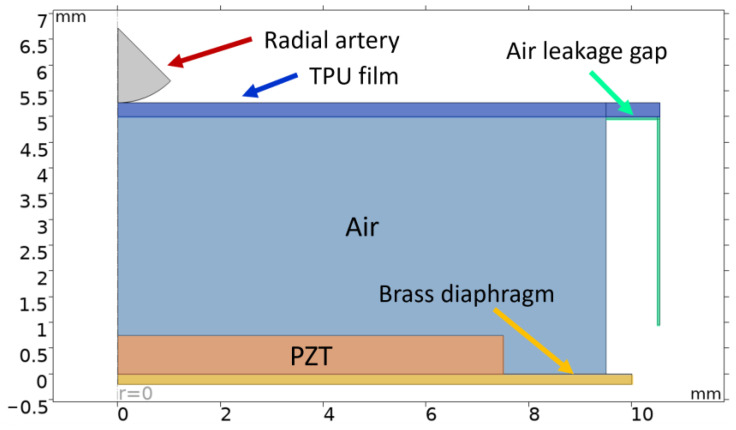
Computational domain.

In order to simulate the behavior of the platform, a two-dimensional axisymmetric computational model was developed in COMSOL Multiphysics. A computational domain was built around a commercially available piezoelectric disc (Figure 2). This PENG consists of a PZT-5H ceramic that is deposited on the top of a brass diaphragm. The space in between the PENG and an elastic film is filled with air. The elastic film is made from 3D-printed thermoplastic polyurethane (TPU). Right near the TPU film, there is a thin air gap domain which allows air to flow in and out of the chamber during the compression and expansion of the film. The air leakage gap is introduced to account for and model the non-ideal sealing of the chamber. A circular domain on the top represents the radial artery. The volume that is present on the back side of the PENG is assumed to be large enough, so its air compressibility does not influence the output results and thus it is not present in the computational domain.

The mathematical model which is employed in COMSOL Multiphysics solves the governing Navier–Stokes equations. The flow is considered to be isothermal, thus omitting calculation of the energy equation. The momentum Equation (1) in differential form for compressible fluid flow:

(3) $$\rho \left(\frac{\partial u}{\partial t}+u\cdot \nabla u\right)=-\nabla p+\nabla \cdot \left(\mu \left(\nabla u+{\left(\nabla u\right)}^{T}\right)-\frac{2}{3}\mu \left(\nabla \cdot u\right)I\right)+F$$

where ρ is the air density (kg/m^3^), u is the flow velocity vector field (m/s), p is the pressure (Pa), μ is the air dynamic viscosity (Pa·s), **I** is the identity tensor and **F** is the force vector field that is translated from the film (N).

Due to the slow velocity and laminar nature of the flow at the particular dimensions and rate of deformation of the film, the creeping flow assumption can be made. This way, inertial forces can be assumed to be non-existent, and thus (3) yields:

(4) $$\rho \left(\frac{\partial u}{\partial t}+u\cdot \nabla u\right)=0$$

(5) $$∴0=-\nabla p+\nabla \cdot \left(\mu \left(\nabla u+{\left(\nabla u\right)}^{T}\right)-\frac{2}{3}\mu \left(\nabla \cdot u\right)I\right)+F$$

The momentum Equation (5) is solved in conjunction with the continuity equation, which for a time-dependent study has the form:

(6) $$\frac{\partial \rho }{\partial t}+\nabla \cdot \left(\rho u\right)=0$$

The load that is imposed on the top surface of the piezoelectric disc is a result of pressure and viscous forces acting together:

(7) $$F=-n\cdot (-\nabla p+\nabla \cdot \left(\mu \left(\nabla u+{\left(\nabla u\right)}^{T}\right)\right))$$

where **n** is the normal vector to the piezoelectric disc.

**Figure 3:**
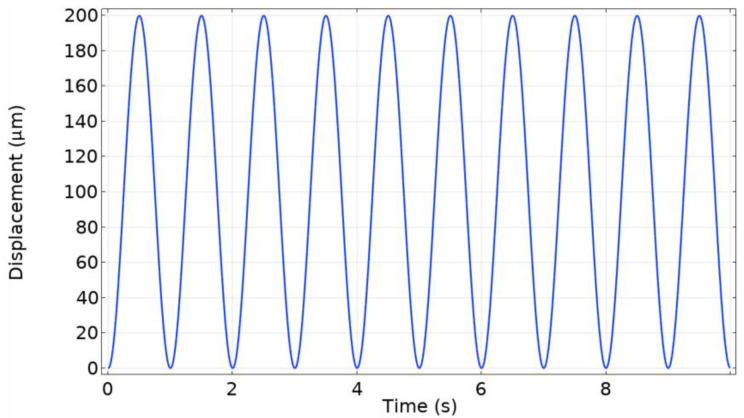
A baseline sinusoidal radial artery displacement signal.

The computational model that was developed works in a simple manner: the radial artery domain travels along the z-axis and presses against the TPU film. The deformation which is caused compresses the air inside the chamber and this in turn causes the pressure to rise. The rise and fall in the pressure inside the domain acts upon the PENG surface, which reacts to it by producing a proportional amount of voltage. The prescribed displacement waveform was a periodic sinusoidal signal at a frequency of 60 BPM (1 Hz) (Figure 3). The signal amplitude, which dictates how far the radial artery domain travels, was selected to be 100 μm since the radial artery can undergo a diameter change of up to 200 μm [30]. Thus, in the model there is a coupling of solid mechanics, fluid–structure interaction and piezoelectricity interfaces.

**Figure 4:**
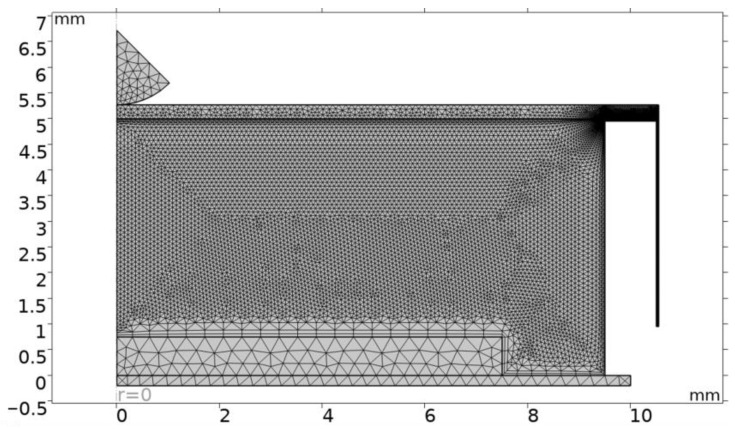
A computational mesh.

The computational mesh consists of triangular and rectangular elements (Figure 4). The air channel is a region of significant fluid flow gradients, and thus an extremely fine rectangular mesh (9984 elements) is located in this region. Moreover, the mesh is denser in the region closer to the boundary where the air enters the chamber. Owing to the higher degree of deformation, the chamber and the TPU film domains are meshed using a finer mesh (8716 elements, minimum size of 0.00104 mm and maximum size of 0.09 mm with a growth rate of 1.08) as compared to the PENG domain (233 elements, minimum size of 0.0546 mm and maximum size of 0.365 mm with a growth rate of 1.13), which is a stationary solid body and its structural analysis does not require a demanding resolution.

The duration of simulations was set equal to 10 s with a timestep of 0.15 s using a coupled solver, which is available in COMSOL. An initial study was carried out on the design that corresponds to the so-called “baseline case”. Subsequently, the effect of the following parameters was studied:

- Frequency of the pulsations
- Width of the air channel
- Height of the air channel
- Thickness of the TPU film
- Elastic properties of the TPU film
- Type of the imposed signal (Sinusoidal and Windkessel waveforms).

## 3. Results and Discussion

**Figure 5:**
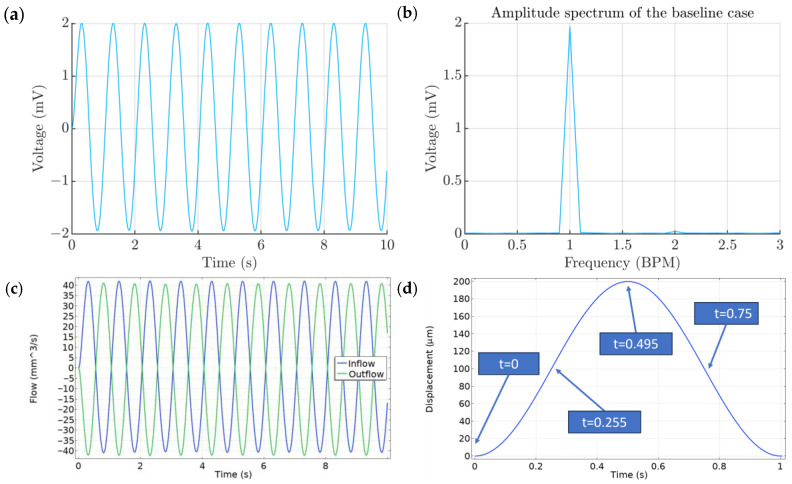
(**a**) Baseline case. Voltage output of the PENG. (**b**) Spectrum of the output voltage. (**c**) Flow rate plot. (**d**) Phases of the model.

The baseline case, which is depicted in Figure 2, assumes that the air channel width is equal to 2.5% of the chamber diameter, the air channel height is equal to 5 mm and the radial artery displacement occurs sinusoidally at a rate of 60 BPM. The voltage output of the model reaches a maximum value of 1.97 mV (Figure 5a). The FFT analysis of the voltage output (Figure 5b) corresponds to an almost perfect sinusoidal signal while having a small multiple at 120 BPMs. The inflow and outflow plot (Figure 5c) shows the amount of air that moves in and out of the chamber during each simulated cardiac cycle, with a maximum rate of 40 μL/s.

**Figure 6:**
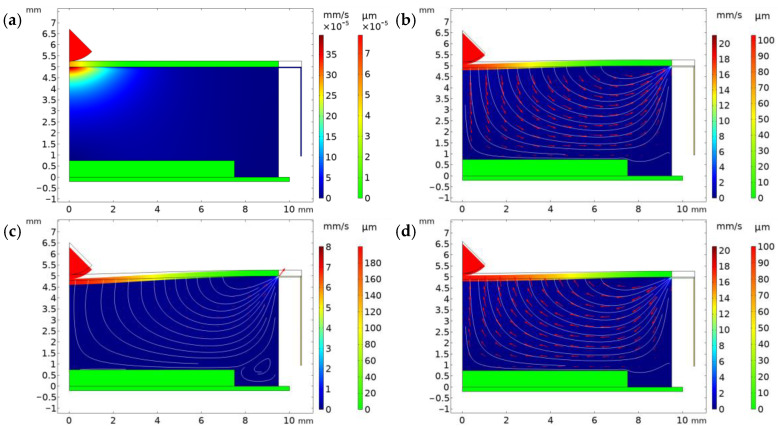
(**a**) The velocity plot at t = 0. (**b**) The velocity plot at t = 0.255. (**c**) The velocity plot at t = 0.495. (**d**) The velocity plot at t = 0.75.

A full cycle of the chamber can be divided into four main phases, which are presented in Figure 5d: initial undeformed state, radial artery expansion, maximum deformation state and radial artery contraction. At first, the air, which is located inside the chamber, is at a resting state and the TPU film is not deformed (Figure 6a). When the radial artery starts expanding, it raises the pressure, which in turn causes air in the chamber to press against the PENG, while simultaneously a small amount of air escapes from the chamber through the thin air channel (Figure 6b). The velocity vector plot of Figure 6c shows that the film deformation causes the air to form a vortex, which initiates right near the point of contact. When the radial artery reaches its maximum size (Figure 6c), the chamber is at rest once again since there is no more variation in pressure. During the pressure release, effects opposite to the ones described above take place. The air enters the chamber as the pressure reduces and the PENG returns back into the chamber by radial artery contraction, thus ending the cycle (Figure 6d). Voltage peaks correspond to points where the radial artery domain travels or expands at maximum velocity. The pressure variation and the consequent piezoelectric voltage can be seen as a derivative of the displacement function.

**Figure 7:**
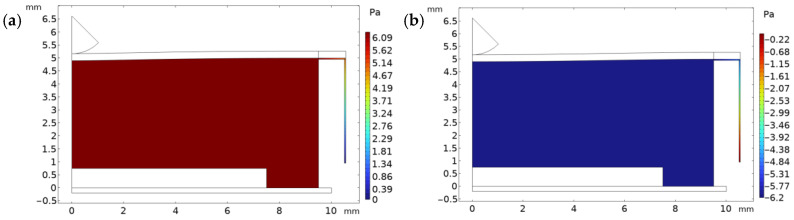
(**a**) Pressure distribution at t = 0.255. (**b**) Pressure distribution plot at t = 0.75.

The pressure plot shows the exact areas where values of high pressure are concentrated. During the radial artery expansion and contraction, the chamber is pressurized uniformly (Figure 7a,b), while a gradient is present at the air leakage channel due to the very high resistance associated with its very small size. For both expansion and maximum deformation, the gradient was found to be the same.

The signal that was imposed on the radial artery domain could be enhanced in order to become more representative of the physical pressure variation occurring inside arteries. The radial artery waveform can be modeled via the so-called Windkessel model, also known as the 0-D model, which is an electronic–hydraulic analogy used for modeling hemodynamics of cardiovascular systems [31]. In this case, the resistance of the blood flow going through arteries and capillaries is analogous to electrical ohmic resistance, the capacitance (F) is called compliance and it accounts for the elastic properties of blood vessels and the inductance (H) is called inertance and describes inertia in the blood flow. Finally, the impedance describes how blood flow changes with a change in heart rate and, similarly to the transmission line, it can be represented as a separate model element. The combination of all these components allows us to accurately reconstruct a waveform in a given segment or segments of the arterial tree. These parameters can be tuned to make the model output waveform personalized.

**Figure 8:**
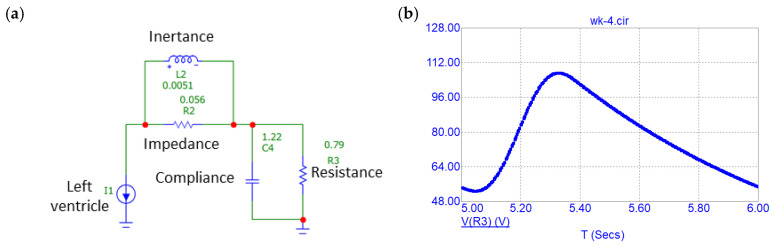
(**a**) The Windkessel lumped model. (**b**) The pressure output of the Windkessel model.

In the present work, a simple 4-element Windkessel model for simulating aortic pressure [32] is employed and is presented in Figure 8a. The circuit was built in the circuit analysis software Micro-Cap 12 and simulated for a duration of 16 s, out of which the first 6 s, which correspond to the charging of the capacitor, were excluded. In this circuit, a current source was used to mimic how the left ventricle is supplying blood flow to the aorta. The voltage output of the circuit, which is analogous to blood pressure, was acquired from the R2 resistor of Figure 8b and afterwards it was normalized. The normalized signal was imported into COMSOL and used to prescribe the displacement of the artery as a boundary condition of the outer surface of the TPU film.

**Figure 9:**
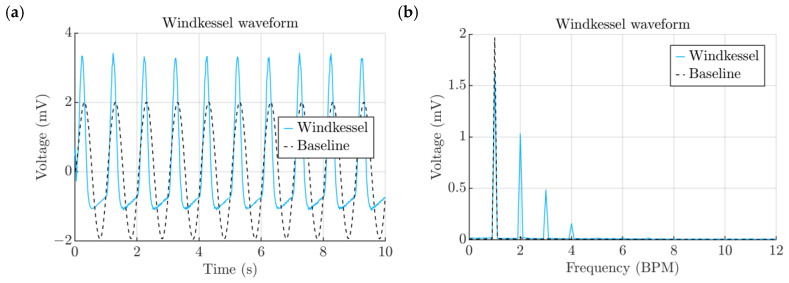
(**a**) Effect of the Windkessel signal. (**b**) Spectra of signals obtained from the Windkessel comparison study.

A simulation was carried out using the Windkessel waveform as the forcing function and was compared with the analysis that was performed using the sinusoidal waveform (Figure 9a). The overall behavior of the harvester was quite similar in both cases, but the maximum and minimum voltage output values differed. The major difference between the two signals can be discerned in their spectra (Figure 9b). As opposed to the sinusoidal waveform, the complex nature of the aortic waveform resulted in multiple noticeable peaks over 120, 180 and 240 BPMs. Most noticeably, the peak at 60 BPM is less than the one corresponding to the sinusoidal waveform; however, with the addition of other multiples, the aortic waveform not only carries more energy, but it also distributes it along different maximum and minimum values, which is important from the circuit design point of view.

**Figure 10:**
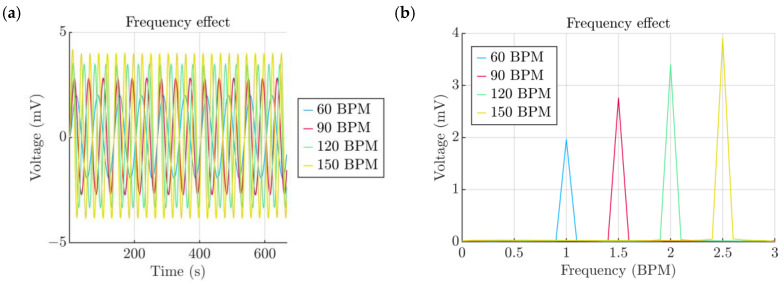
(**a**) Effect of the actuation frequency. (**b**) Spectra of signals obtained from the actuation frequency study.

Subsequently, the influence of the signal frequency was studied. The simulated range varied from 60 BPMs (1 Hz) to 150 BPMs (2.5 Hz), with an increment of 30 BPMs (0.5 Hz). Similarly to the results obtained in our previous work [33], due to the inherent impedance of the PENG, a higher frequency of actuation led to a higher voltage output, as it is illustrated in Figure 10a. The TPU film material properties appeared not to have a tangible effect on the performance. The amplitude of the 60 BPM baseline case was lower compared to 3.9 mV at 150 BPMs, which was almost 2 mV higher (Figure 10b). This influence in conjunction with the output of the Windkessel model may suggest that the energy conditioning circuits can be tuned to frequencies that are multiples of the carrying frequency.

**Figure 11:**
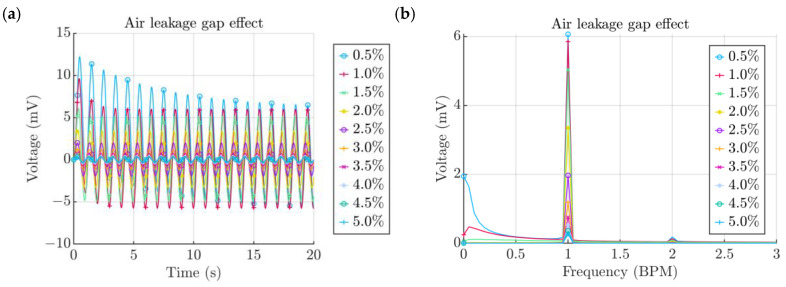
(**a**) Effect of the air leakage gap. (**b**) Spectra of signals obtained from the air leakage gap study.

As the width of the air leakage gap cannot be precisely controlled, there is always going to be a certain tolerance that the enclosure and the TPU cap abide to. Since the TPU is an elastic material, it is possible to account for the gap via reducing the diameter of the cap. Having a tighter fit decreased the air leakage gap width and made the energy harvesting process more efficient (Figure 11a). The transient process that was present for smaller air leakage gaps, and is indicated by the initial high amplitude values, were subsequently stabilized. This could be explained by the chamber not having time to completely de-pressurize after a cycle is completed before the start of the following one. Therefore, the residual pressure was carried on from cycle to cycle until it was fully relaxed. From the spectra it can be seen that the voltage output increases each time the gap width is decreased by 0.5%. However, the most noticeable and efficient of these are the changes from 2.5% to 2% and from 2% to 1.5% (Figure 11b). Achieving a better sealing percentage, below 1%, did not appear to offer a tangible increment.

**Figure 12:**
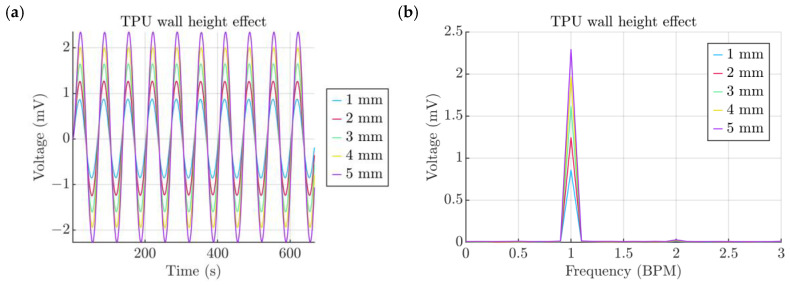
(**a**) Effect of the TPU wall height. (**b**) Spectra of signals obtained from the TPU wall height study.

Another way of increasing the air leakage gap resistance is to make the TPU cap walls longer along the lateral side of the enclosure. In this way, the air has to travel a longer distance, which increases the pressure inside the chamber. The results served as a proof of this concept and showed (Figure 12a) that the increase in the TPU baseline wall length from 4 mm to 5 mm increased the voltage output to 2.29 mV and, the shortest wall, which barely provides grip at all, provided an output of 0.86 mV (Figure 12b). Interestingly enough, there is also a, similar to the leakage gap, trend where the gain from the wall extension slowly degrades with each increment.

**Figure 13:**
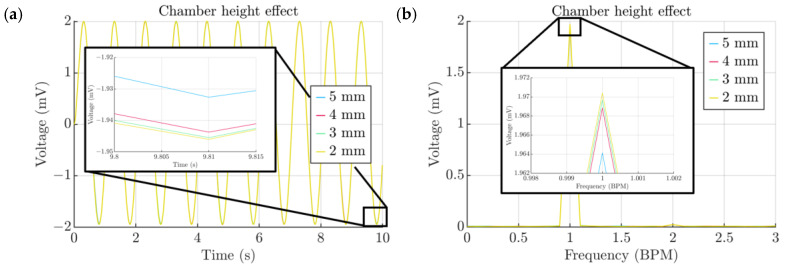
(**a**) Effect of the chamber height. (**b**) Spectra of signals obtained from the chamber height study.

The chamber height parameter was investigated and its interdependence with the voltage output was established. As a result, the decrease in chamber height from 5 mm to 4 mm had a very small influence on the voltage output of the order of 4 μV and all further decrements had about a 1 μV increase in voltage (Figure 13a,b). From a design point of view, it means that it is possible to decrease the chamber height without disturbing the overall behavior of the platform, and at the same time also achieve a slimmer appearance.

The *Prescribed Displacement* of the radial artery domain could be alternatively swapped with a *Boundary Load* to study the elastic properties of the TPU film. In this case, the deformation that the chamber and the film domains undergo are based on the force applied to the TPU film. The load can be imposed on the top surface of the film and follow the same baseline sinusoidal waveform. The TPU material properties are listed in Table 1.

**Figure 14:**
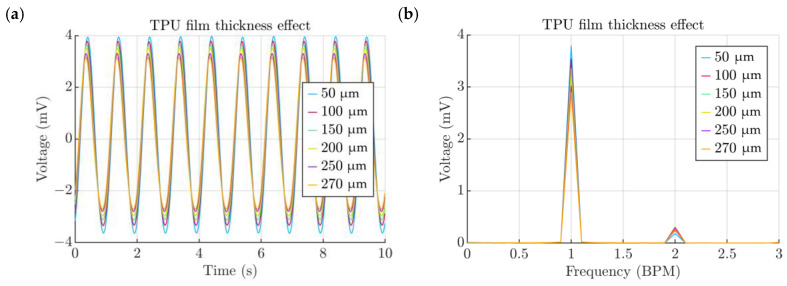
(**a**) Effect of the TPU film thickness. (**b**) Spectra of signals obtained from the TPU film thickness study.

The thickness of the TPU has a direct impact on the performance of the energy harvester and should be kept as low as possible, as in this way the TPU film elastic properties oppose the cardiovascular pressure to a lesser extent. The plots show that the baseline thickness of 270 μm produced 2.90 mV and that 50 μm could produce up to 3.79 mV (Figure 14a,b).

**Figure 15:**
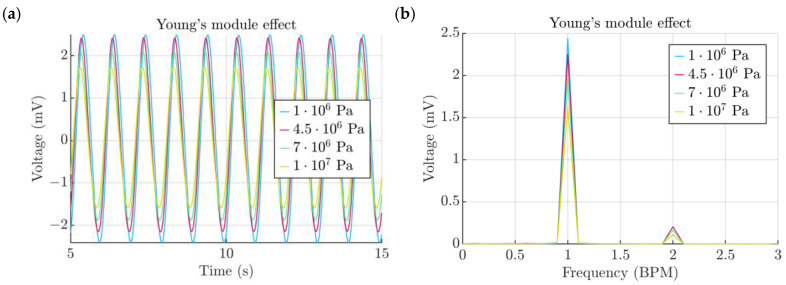
(**a**) Effect of chamber height. (**b**) Spectra of signals obtained from the chamber height study.

The exact elastic properties of a given TPU film might vary depending on factors such as the nozzle temperature, air moisture content, the adhesive strength of each individual line, the line pattern and the type of the TPU material. Therefore, it is important to capture the Young’s module effects on the voltage output. The baseline value of 4.5 MPa was compared to a range of values varying from 1 to 10 MPa (Figure 15a). The obtained results may serve as a design reference information and can be used to find the value of the Young’s module of a particular film (Figure 15a).

## 4. Experimental Results and Discussion

### 4.1. Design and Fabrication of the Harvester Enclosure

**Figure 16:**
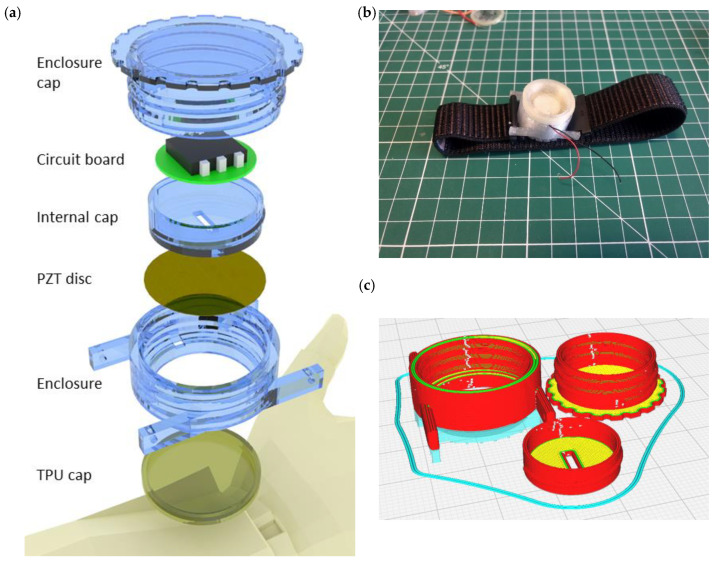
(**a**) The energy harvesting platform assembly. (**b**) The manufactured prototype. (**c**) Sliced PET parts.

The harvester enclosure consists of four parts: a case, a cap, an insert and a TPU film (Figure 16a). The film was made out of BASF Forward AM Ultrafuse TPU 80A LF, whereas all other parts were manufactured from transparent UltraFuse PET filament. The insert is envisioned to accommodate on-board electronics like a power management unit and/or a biosensing hardware platform. The cap has threading both inside and outside; thus, the insert can be mounted inside the cap and the cap can be screwed on the top of the case. The case represents a harness for the PENG and acts as a point of attachment to the body. To firmly fix the PENG in place, the cap wall, which also serves as a base for inner and outer threading, is extended in a way that when the cap is fully inside, it presses against the brass diaphragm of the PENG. This helps to seal the design and minimize the use of glue or any other sealant. The assembled prototype platform is then equipped with a wrist strap and is positioned at the top of the radial artery (Figure 16b).

A fused filament fabrication method was employed to make a prototype. The slicing of parts was made in Cura 5.0 (Figure 16c) and an Ultimaker S5 3D printer was used for the fabrication. Apart from the case, other parts do not require print support and the case can be printed with the support made from the same material as the case. No adhesives or brims were incorporated for printing. Print settings that were used for the PET parts were based entirely on recommended settings by UltraFuse (Table 2).

As it can be seen from the simulation results, the elastic film has a paramount importance on the harvester’s performance. Thus, it is imperative to be able to accomplish optimal thickness and reproducibility of the 3D-printed film.

By design, the TPU cap consists of a film which is formed only by the initial layer of filament and walls that tightly fit the respective connector walls of the enclosure case. Owing to the elastic properties of the TPU filament, the diameter of the cap could be varied to tightly fit the enclosure, thus accounting for tolerances between connecting parts and reducing the width of the air gap. Because the fit can be varied, the use of glue is not necessary.

The thickness of the TPU film can be modified by controlling the initial layer height parameter. It was possible to gradually decrease the thickness of the TPU film from 270 μm (the default recommended value of the initial layer height) down to 100 μm. In order to achieve this, certain parameters such as line width, the values of print and build plate temperatures were optimized (Table 3).

A smaller line width allowed us to fill the film with much more filament lines, improving the flexibility, whereas the bed temperature provided better adhesion in between extruded filament lines. The printing temperature was proportionally increased to suit the printing bed temperature, thus reducing the heat gradient between the build plate and the nozzle. Failing to achieve the set parameters resulted in an inability to peel the cap off the build plate for the 100 μm thickness, as it started ripping off either in the middle or near the edges. As a tradeoff, the 100 μm thickness had an issue with its film getting stretched while being peeled off. The issue was later dealt with by reducing the diameter of the cap by 1 mm, thus achieving a tight fit with the enclosure which also allowed the film to remain stretched due to tension.

**Figure 17:**
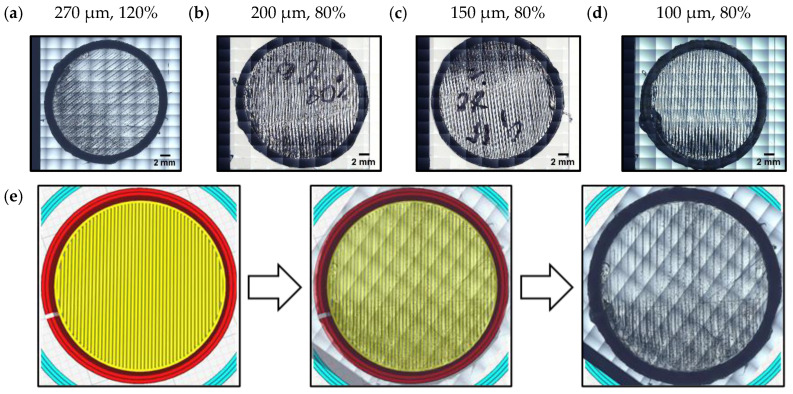
(**a**) A 270 μm 120% TPU film. (**b**) A 200 μm 80% TPU film. (**c**) A 150 μm 80% TPU film. (**d**) A 100 μm 80% TPU film. (**e**) Comparison between the sliced model and the manufactured 270 μm film.

Given the optimized parameters, the 100 μm TPU film became possible to manufacture. An initial layer line width of 120% was used for printing a 270 μm film while other thicknesses used an 80%-line width (Figure 17a–d). A Nikon Microphot-FX light Microscope with 4× magnification was employed to obtain mosaic images of films for the purpose of quality control. While not apparent, the individual filament lines could be distinguished, especially when compared to a Cura 5.0 sliced model image (Figure 17e). The transparency of the film also served as indirect evidence of the thickness, as a more transparent film indicates that it is also thinner.

**Figure 18:**
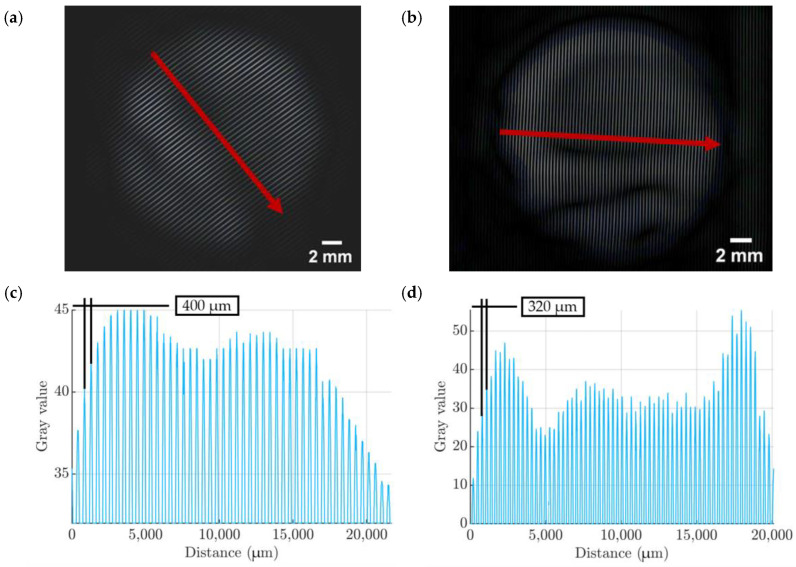
(**a**) Inverse-FFT of the 270 μm film with a surface plot line. (**b**) Inverse-FFT of the 100 μm film with the surface plot vector. (**c**) A surface line plot for the 270 μm film. (**d**) A surface line plot for the 100 μm film.

The ImageJ 1.54g software [34] was used to assess the width of the films in a qualitative way. A combination of FFT and inverse-FFT of an image were used to obtain spectra of 120% 270 μm and 80% 100 μm films. Subsequently, a single line was placed in the image (Figure 18a,b), and its profile was plotted (Figure 18c,d). The distance between peaks corresponds to the initial layer line width parameter. The advantage of using this method over a simple visual inspection is that inverse-FFT averages the symmetry found in the image and provides a much better contrast. Moreover, any artefacts, such as the exact places of films getting bent (Figure 18a,b), could be easily identified.

### 4.2. Acquisition of Physiological Data

**Figure 19:**
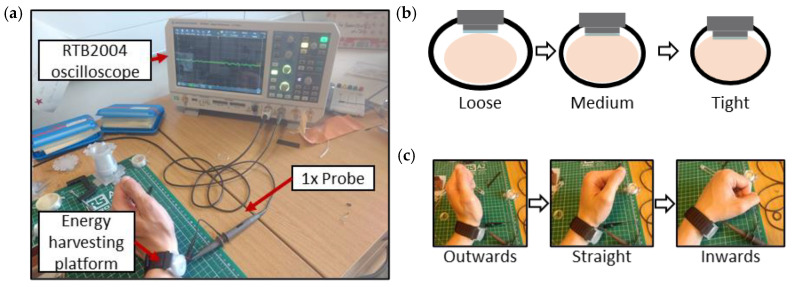
(**a**) Experimental setup. (**b**) Studied strap fits. (**c**) Studied wrist positions.

In order to obtain real physiological data, the fabricated piezoelectric energy harvesting platform was placed on a left-arm wrist, right on top of the radial artery. The PENG was connected to a Rhode & Schwartz RTB2004 oscilloscope via a 1× attenuation probe (Figure 19a). Each signal was recorded for a duration of 60 s and contained 129,864 data points. The studied parameters were strap circumference (Figure 19b), wrist angle position (Figure 19c) and TPU film thickness. The baseline case comprised a 270 μm TPU film with a tight fit and outwards wrist position. The results comprised the original signals, which were filtered with a low-pass filter to cut-off any noise higher than 720 BPM (12 Hz), and the combined amplitude spectra for the given parameter. The spectra are given in both the original form and with a Savitzky–Golay filter applied for easier comparison.

Because most fitness trackers, smartwatches and wearable biosensors use straps, the relationship between how hard the harvester is pressed against the wrist was investigated. There were three distinct fits: loose, medium and tight. Due to physiological differences between individuals, these general terms were used to assess the fit of the strap based on the descriptions of Table 4.

**Figure 20:**
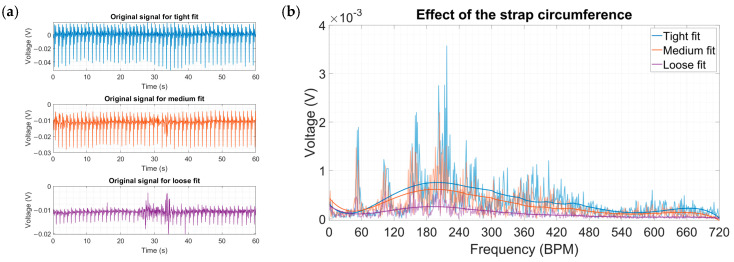
(**a**) Original signals for the three different strap fits. (**b**) Amplitude spectra for the three different strap circumferences.

The “Tight” fit was selected as the baseline fit for the rest of parameters due to the better quality of data acquisition. The results showed that the selected range of fits had almost a linear relationship (Figure 20a,b). The “Loose” fit showed the lowest voltage output results, yet the radial artery waveform could be distinguished. The “Medium” fit had better output and ended up being almost three-times better than the loose fit. The “Tight” fit had the best voltage output, topping at 3.57 mV at 200 BPMs.

**Figure 21:**
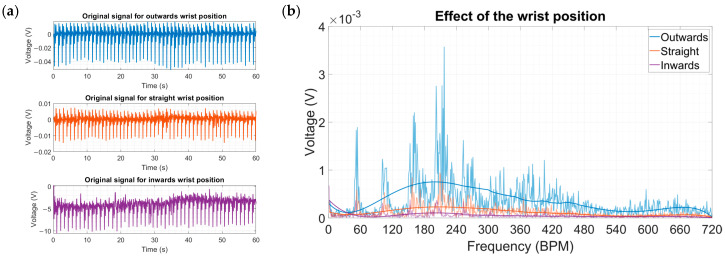
(**a**) Original signals for the three different wrist positions. (**b**) Amplitude spectra for the three different wrist positions.

The effect of the wrist angle position was investigated (Figure 21a,b). When the hand is pointing inwards to the body, the muscle strain is minimal, and the radial artery was positioned far from the energy harvester contact point. Conversely, when the wrist is pointing outwards, the radial artery could easily be pushed against the radius bone; the pulsations were much more prevalent and thus it was selected as the baseline parameter for the experiment. The empirical data showed that there were noticeable differences between all three studied positions. The inwards position waveform was almost close to the detection limit of the oscilloscope and the straight position showed incremental improvement yet still fell short of reaching reasonable output levels. Positioning the hand outwards helped to scavenge the most energy, which was 3.5-times more than the straight positioning. From the results, it can be deduced that while a person wears the energy harvester as a wristwatch, the amount of energy is going to vary significantly, especially considering that the outwards position is less natural than the other two options.

**Figure 22:**
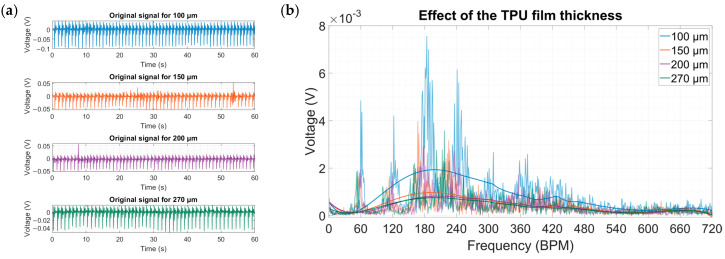
(**a**) Original signals for the three different TPU film thickness values. (**b**) Amplitude spectra for the three different TPU film thickness values.

Lastly, the fabricated range of TPU films was tested with the help of the prototype microdevice. As it can be observed from the results, the trend set by simulations agreed with the experimental data (Figure 22a,b). The baseline 270 μm yielded a maximum voltage of 3.57 mV at 200 BPMs. The intermediate thickness values showed small changes, which is especially apparent when looking at the filtered signals. However, the 100 μm film demonstrated a significant increase in the output, up to 7.55 mV at 180 BPMs. It might be related to the decrease in the cap diameter, which provides a better sealing for the cap.

**Figure 23:**
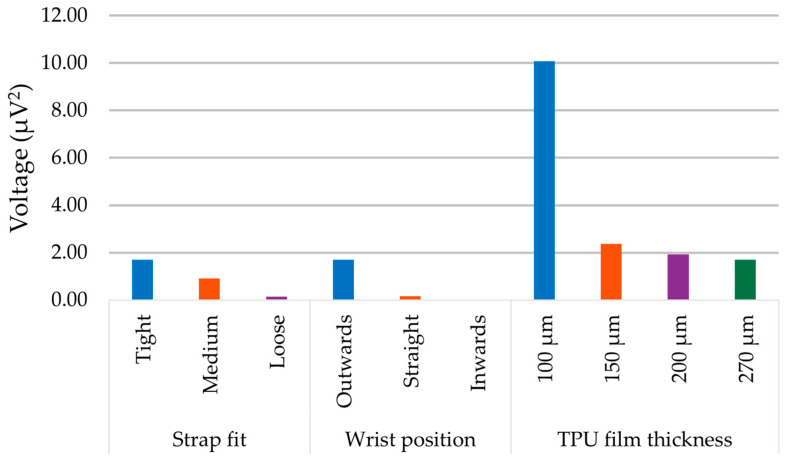
Comparative results from the parametric study.

It is possible to provide an estimate of power that each signal carries by computing the area under the curve of the given amplitude spectrum (Figure 23). The diagram illustrates a summary of the obtained empirical data and provides a quantitative assessment of the methods used to increase the power output. The different colours correspond to the parametric settings and are the same with the colours that were used in Figure 20, Figure 21 and Figure 22. 

**Figure 24:**
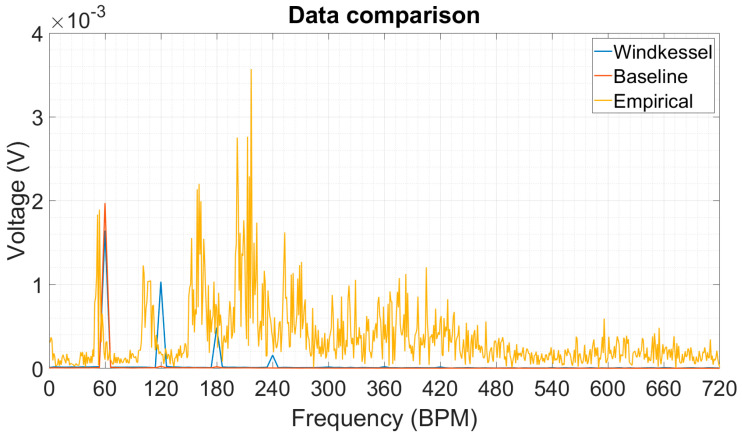
Comparison between the simulated and acquired signals.

A more general finding is that the actual physiological signal has a significant amount of energy distributed along multiples of the carrying frequency, which are 120, 180 and 240 BPMs (Figure 24). Moreover, the Windkessel signal resulted in four distinctive frequencies with a gradually reducing signal amplitude, which is an opposite trend compared to the empirically obtained signal which had two additional identifiable multiples where the third and fourth multiples had the highest amplitudes. The explanation of this behavior might be related to the resonances happening inside the enclosure, the hand tremor and the inherent complexity of the real pulse waveform shape. Overall, there is a really small deviation from the Windkessel signal and the actual radial artery waveform at 60 and 120 BPMs; however, the third and fourth multiples differ significantly. This trend suggests that when scavenging energy from the radial artery with the given platform, the power management unit can be more easily integrated into the system since the output has a relatively high frequency and proportionally high output as compared to the signal that is present at 60 BPMs.

Further work on hybridization is currently underway. The space between the PENG and the internal cap (Figure 15a) is envisioned to be used as a base for reverse electrowetting on a dielectric (REWOD) electrostatic energy generator; and, in this way, the displacement of the radial artery is going to be used by both the PENG and REWOD together. In our previous work [33], a computational study on perspectives of using REWOD with the radial artery displacement was conducted and the next step of the ongoing research is to hybridize two energy harvesting principles into a single package.

## 5. Conclusions

A 3D-printed energy harvesting platform was designed and investigated through a combination of numerical and experimental approaches. A COMSOL Multiphysics mathematical model was designed and a series of parametric studies were performed in order to assess its energy harvesting performance. The 4-element Windkessel model was utilized to describe the arterial pressure variation and the simulation results were compared against those from the baseline sinusoidal signal. The air leakage gap was taken into account and the underlying dependency between the output and the gap width value was established. The height of the TPU film showed a direct relation to the performance of the PENG, whereas the height of the chamber showed a negligible influence. The elastic properties were studied and design reference plots were produced. The prototype of the energy harvesting platform was manufactured using the FFF method. The TPU film print settings of a 3D printer were optimized to reduce the thickness from 270 μm to 100 μm and a method of quality control of the produced film was presented. The prototype was used for physiological data acquisition and the effect of parameters such as the TPU film thickness, the wrist angle and the strap circumference on energy harvesting were studied. The experimental results showed that the wrist angle and the strap circumference are significantly related to the output and that the best results could be achieved in platform positions that are usually not natural for wearing. The acquired empirical signal spectrum showed that the energy contained within a signal was spread along the same frequencies as the Windkessel signal, yet with different frequencies having the highest output.

## Figures and Tables

**Table 1:** TPU material properties.

Property	Variable	Value	Unit
Density	rho	1100	kg/m^3^
Young’s modulus	E	4.5	kPa
Poisson’s ratio	nu	0.40	1

**Table 2:** PET print settings.

Parameter	Value
Print core	0.4 mm
Line width	120%
Print temperature	220.0 (initial layer: 215.0) °C
Bed temperature	65.0 (initial layer: 70.0) °C
Speed	60.0 mm/s
Initial layer height	270 μm

**Table 3:** TPU print settings.

Parameter	Default Value	Optimized Value
Print core	0.4 mm	0.4 mm
Initial layer line width	120%	80%
Print temperature	235 (initial layer: 233.0) °C	243.0 (initial layer: 235.0) °C
Build plate temperature	60.0 (initial layer: 55.0) °C	70.0 (initial layer: 65.0) °C
Print speed	25.0 mm/s	25.0 mm/s
Initial layer height	270 μm	100 μm

**Table 4:** Descriptions of the different harvester wrist fits.

“Loose”	the harvester was gently pressed against the skin with quite significant play in between the strap and the wrist
“Medium”	a comfortable fit that fixed the harvester in place yet allowed it to have some movement, akin to how a watch or a fitness tracker would be worn
“Tight”	the harvester did not have any play and was firmly pressed against the wrist, a borderline fit which did not induce pain

## Data Availability

The reported data in this review article are obtained from the references which are included in this paper.
